# Lectures on Subjects Connected with Clinical Medicine

**Published:** 1836-10

**Authors:** 


### PART SECOND.

### Bibliographical Notices.

---

Art. I.—*Lectures on Subjects connected with Clinical Medicine.* By P. M. Latham, m.d., Fellow of the Royal College of Physicians, and Physician to St. Bartholomew's Hospital.—*London,* 1836. 8vo. pp. 322.

There is much to approve of both in the design and the execution of these lectures. Dr. Latham is one of several distinguished physicians attached to public hospitals in the metropolis, whom the evident inutility, as far as the pupils were concerned, of being followed round the wards by a crowd of uninstructed youths, to whom, as was the old system, nothing was explained, has induced to assist the learners by clinical lectures on medicine. Until of late years, no department of study was so much neglected as that of theoretical and practical medicine. The young men resorting to London were laudably anxious for anatomical knowledge; they were influenced with much zeal as regarded surgical operations; but medicine formed no part of their plan. Nor was this at all to be wondered at. From the earliest days of their apprenticeship, they had entered on the cure of medical cases without scruple, from pains in the side to the worst forms of fever; but of surgical responsibilities they had imbibed a considerable apprehension. Bleeding and tooth-drawing they had daily performed; but they knew that the more important operations were eschewed in country places, and transferred to the surgeons of the county hospital, whose anatomy had been kept up, and whose hands were exercised. Even after a year or two passed in London, their ideas on these subjects remained unchanged. The accomplished youth returned to his province, and his talk was of Cline, or of Astley Cooper, or of Abernethy,—great men, indeed, and well deserving of his grateful mention; but, of the practice of the hospitals of London in fevers, or in inflammations, or in chronic disorders, no one ever heard a word; for of these things the young surgeon had taken no kind of notice. Yet it is well known that his daily duties, from the very day of his commencing practice, were chiefly medical, and that his life might be passed without his ever being intrusted with a capital operation. If rash and presuming, he proceeded fearlessly to treat disorders of the progress of which he was ignorant, to the no small detriment of his patients; or, if diffident and conscientious, suffered many anxieties for many years. We suspect that much of the blame of this was attributable to the incommunicativeness of the hospital physicians. Surgical diseases almost explained themselves, or were explained by a few striking observations. The medical cases were obscure, and the older physicians are supposed to have set some store upon obscurity, as enhancing that talent of which they knew the public were ill fitted to judge.

It cannot, therefore, be wondered at that medical practice remained so long imperfect, or that even, at this day, with all the advantage of clinical teachers, the practice of physic should be so singularly unscientific; and that imperfect diagnosis, excessive bleedings, and the extravagant administration of purgative medicines, should be so characteristic of English medicine. A teacher, therefore, who endeavours, as Dr. Latham has done, to suggest to the pupil how to observe and what to observe, does a very essential service to the school to which he belongs.

Dr. Latham has in a particular manner directed attention to the value of symptoms, those more especially which are attained to by auscultation, and he has attempted to simplify the practice of auscultation, with a view to increasing its popularity and its general applicability. As we are convinced that not one student in ten leaves the London schools enabled to avail himself of the uses either of percussion or auscultation, we attach considerable value to this part of Dr. Latham's labours. Practitioners are ever prescribing for the names of diseases. With many of them, every pain is an inflammation, every palpitation a disease of the heart, every form of difficult breathing an asthma or a dropsy; whilst a large proportion of obscure diseases are ascribed on small grounds to some undefined state of the stomach. The debility attendant on fever is, we know, still not unfrequently treated by tonics or stimulants, and numerous organic diseases are derided as nervous, and exasperated by tonics from the mineral kingdom. We speak of things which we daily witness; and the effect on our minds is the growing-up of a belief, to which every year adds strength, that not a few invalids are annually destroyed by mal-practice; for which, if there is no moral excuse, there is unfortunately no legal punishment.

In Dr. Latham's first lecture, we are rather disposed to object that he has said too much in depreciation of the acquirements of a physician or surgeon. It may be true that too much is sometimes said in introductory lectures concerning the variety of accomplishments essential to the practitioner; but this parade, as far as London is concerned, has, we imagine, been very harmless, seldom leading to an inconvenient accumulation of general knowledge. If the pomp and pretence of learning, conjoined with real ignorance, furnished matter of just ridicule to Molière and Le Sage, as Dr. Latham truly enough observes, the coarse and illiterate ignorance of many young men who are licensed as surgeons has materially degraded the profession of physic, and contributed to drive many of the educated classes of the community into the hands of fraudulent quacks, who, to the advantage of a decent general education, adding consummate knowledge of mankind, were enabled to dispense with medical knowledge and honesty. We apprehend, too, from the concluding passages of Dr. Latham's first prelection, that his prejudices lean to the college side of education, and indispose him to the belief that the years now lost at public schools, and in the grammar schools which imitate their faults, might be far more profitably spent, and admit of an acquisition of scientific knowledge not far short of that set forth by certain lecturers as requisite to the efficient study of medicine. A knowledge of chemistry, of botany, and of certain branches of natural philosophy, and an acquaintance with one or two of the modern languages, might easily be acquired by any youth in his days of school learning and of apprenticeship, and these would prepare him for his professional studies, and allow him to devote his time and mind to them when he began to attend lectures and hospital practice. But he too generally enters on his professional studies ignorant of every modern language but his own, and very imperfectly skilled in that; devoid of science, and only slightly tinged with the Latin grammar. Happily, every year is adding to the resources of country towns with respect to general and scientific education: the effectual reformation of the decayed grammar schools must soon take place, and societies in aid of all liberal knowledge are becoming everywhere instituted.

With the present difficulties in the way of preliminary instruction, we agree with Dr. Latham that the period of medical education is much too short to admit of multifarious studies, and even that such studies interfere with and obstruct the acquirement of indispensable practical knowledge.

Dr. Latham's second lecture is on the subject of Clinical Teaching; which, he truly observes, has been "separated too much from the wards and the bedside, and has deviated into a discussion of abstract pathology and therapeutics." The truth is, that many clinical lectures, falsely so called, are carefully composed in the closet, not suggested in the hospital; and not meant to inform the pupils, but merely to sell. They are an elegant form of advertisement, possessing all the advantages, without the scandal, of certain other advertisements, which point out the proper quarter in which to apply when "men of education and professional skill" are scarce. It is really difficult to conceive an instructive *clinical* lecture fit for publication. We should say of it, as was once said of a political harangue by a great authority, "if it reads well, it was a bad speech." It may be a good lecture, full of interest, full of practical knowledge, abounding with learning, and smartly expressed; but it is not clinical.

There is often, indeed, but small inducement to the lecturer to deliver a strictly clinical lecture. Such a lecture is a commentary on cases; but the cases commented upon must have been seen and watched by the hearers; and Dr. Latham, whose experience as a teacher is considerable, tells us that "five out of six of those who profess to attend the medical practice of this hospital" (St. Bartholomew's,) "never watch a single case of disease through its entire course, during the whole period of their pupilage: I say this," he adds, "with great sorrow, and as a warning to those whose pupilage has yet to begin." We suspect that many of our foreign brethren could form no adequate idea of the degree of carelessness of London students, if unenlightened by this passage of one of their own teachers.

After some highly interesting observations on the views which led him to undertake the office of clinical teacher, or as he aptly terms it, a *demonstrator* of medical facts, Dr. Latham thus details his method.

"You will perceive, then, that with me clinical instruction is, as little as possible, a matter of formal lecture. I will tell you the manner of my proceeding.

"Upon the admission of a patient, my first object is to learn the exact nature of the disease I have to deal with; and this is done by my own observation, and by enquiries to which the patient himself or his friends make answer. This is taking the case.

"Now, in taking the case, I desire always to proceed after a certain method; and, when I am able to pursue that method, all the circumstances which I seek to know unfold themselves naturally and easily, and then it is a simple, agreeable, and interesting employment.

"But often, very often, I am driven from all pretence of method in taking the case. The poor patient is embarrassed by the novelty of his situation, or he is deaf, or his disease incapacitates him; and he hardly understands your questions, and gives you strange answers. Thus things drop out confusedly one after another, and you must be content to accept them as they come, and join them together as you can. But, upon these terms, taking a case becomes a very irksome disagreeable business.

"In taking the case, however, if I am able, I always proceed thus:—

"The patient being placed before me, I ask him no question until I have learnt every thing worthy of remark which my own eyes can inform me of. His physiognomy; his complexion, whether florid, pale, or dusky; the general bulk of his body, whether large and full, or spare and wasted; the condition of particular regions, whether swelled or attenuated; and of the surface, whether there be any eruptions or sores upon it, and what is their character; and, lastly, the power of locomotion, whether he have free use of his limbs or not.

"All these are most important particulars, and we ought to make much of them. There can be no doubt concerning them; they are objects of our own observation, and come to us authenticated by the testimony of our own senses. One step securely ascertained leads to another; and from what we see upon the exterior, we obtain a clue for directing our enquiry to the seat and centre of the disease within. If locomotion be hindered, we look well to the brain and spinal marrow; if there be the livid lip and dusky skin, we scrutinize particularly the condition of the heart and lungs; if the whole body, or some of its parts be attenuated, we examine well the organs of nutrition.

"Having thus learnt all I can with my own eyes, and felt the pulse and seen the tongue, I next proceed, in taking the case, to that further enquiry in which the patient takes a part: and first, I ask him concerning his general sensations, especially whether he be hot or cold; and I endeavour to learn whether his heat or cold occur under conditions which constitute fever.

"Next, I inquire into the state of particular organs, and, beginning with the head, I ask after pain, vertigo, and sense of weight, the sight and the hearing, and sleep and wakefulness. Many of these things are only glanced at, or perhaps passed over altogether, if there be no reason to suspect disease of the brain.

"Then, passing to the chest and respiratory organs, I ask concerning pain, and cough, and expectoration, and the state of the breathing under various conditions of exertion and in different postures of the body; and I learn the force and extent of the heart's pulsation.

"These things are hardly dwelt upon, or soon despatched, if there be no suspicion of disease in the chest, but if there be, not all we can learn by simple enquiry is enough to ascertain its nature. The patient must, moreover, be submitted to the process of auscultation. This process, however, in order to avoid interruption, I postpone until other enquiries are finished.

"Lastly, proceeding to the abdomen, I ask here also concerning pain and uncomfortable sensations—the appetite, the digestion, and the evacuations, their frequency, quantity, and appearance; and then I ascertain with my hand its form and fulness, the possible enlargement of particular viscera, the effusion of fluid, or the existence of pain upon pressure.

"Here the examination of the patient ceases, as to his actual condition; but the history of his complaint remains to be learnt, its origin and progress hitherto, and its probable exciting cause.

"Perhaps it would seem more in the order of nature, and therefore the best method, to take the history first of all. Formerly I used to do so, but I found it practically inconvenient. If you first learn the existing complaint, you know how much of its previous history you will require to illustrate it; but if you first enquire the history, since you do not yet know what it is to illustrate, you cannot tell how much of it you shall want, and must allow the patient to tell what he thinks fit; and since every person's complaint is interesting to himself, he is apt to discourse about it rather too much at large, and too little to edification. Therefore it is that I now always enquire the history last, inverting (if you please) the order of nature; and I take care to make the patient answer express questions rather than leave him to expatiate at his own discretion.

"And now the case is taken and recorded in a book by the clinical clerk, not that I deliver over to be recorded all the circumstances that come in the progress of the examination, but only such a selection of them as may serve to declare the disease, and furnish guidance and direction in the treatment of it.

"The case, I say, is now taken, provided there be no suspicion of disease in the chest. But if there be, the patient must be submitted to the process of auscultation." (P. 47.)

"With auscultation I almost always use percussion; and the results of the one perpetually correct or confirm the results of the other, and strengthen the diagnosis.

"But does all clinical instruction consist in directing the mind how to ascertain mere particulars, whether by auscultation or percussion, or by whatever other method is adopted for their discovery in different organs. No. Clinical instruction is not merely occupied in directing observation to facts, but it assists the mind in estimating their value. Thus, when the record of the case has been read aloud, I admit you to share in my deliberation upon all its particulars, while I endeavour to assort them and bring them together, and make them yield all the light they are capable of throwing upon the nature and seat of the disease. Sometimes I can at once come to a confident diagnosis; and when I can, I at once announce what it is, and give my reasons for it.

"Sometimes, after great pains of enquiry, I am still in the dark; and when I am, I say so, and desire to reserve the case for future examination.

"Sometimes, perhaps most frequently, I feel that I have a tolerably right notion of the complaint, but require some circumstances to be more clearly made out before I can be absolutely certain. And then I state what are the circumstances which give me the notion that I have, and what I still desiderate to bring me to a more confident conclusion.

"The case still remains to be prescribed for. In prescribing, I endeavour to be as simple as possible, to make the indications of treatment as intelligible as possible, and the object I have in view clearly seen.

"The case thus examined, and commented upon, and prescribed for, I then commit to your future observation as a medical study.

"In the future progress of the case I am still present, not to give formal lectures, but to take care that none of the circumstances which continue to develop themselves may fall to the ground profitless; and that you may be tutored by *them*, and not by *me*." (P. 55.)

Dr. Latham has here enumerated the chief points comprehended in clinical study; and, under such a preceptor, it must be the student's own fault if his practical knowledge is not increased. It would be desirable, we think, to require from the more advanced of the pupils that they should occasionally examine the patients, and be questioned as to the grounds of diagnosis, particularly in cases affording clear physical signs to be detected by percussion and auscultation. We meet with so many practitioners who are desirous of the information which they know is to be gained by the use of the stethoscope, yet so unacquainted with its method of application, and so unable to distinguish the different sounds, that we would strongly urge on clinical teachers the necessity of giving a few systematic and elementary lessons on the employment of this instrument to all beginners. The student should begin by applying it to the chests of his companions, to familiarize himself with the natural sounds of respiration and of the heart; he should then be induced to apply it in bronchitis, or other cases affording clear indications, and thus educate his ear to distinguish sounds less easily appreciable. The plan proposed by Dr. Latham, (p. 67,) of conducting examinations in the wards of the hospital, might be very serviceable: the practical knowledge of the pupils would thereby be much increased, and readily ascertained. The mere asking of questions in an examination elicits very uncertain proofs of practical acquirement. Many a student who can recognise a disease is puzzled when called upon to describe it; and, although the symptoms might suggest the employment of proper remedies to his mind, the attempt to enumerate the remedies in the examination-room may occasion him considerable embarrassment. Yet it is to be remembered that such examinations are the only means of ascertaining the student's industry; and this is a matter of very great importance.

We believe there are few more perplexing questions to a lecturer than that which he has to answer almost every day in the session, as to the best text-book of practical medicine. Dr. Latham seems doubtful of the benefit accruing to the student from having any such book; but the student's observation is certainly both stimulated and assisted by frequent reference to good practical works. The work most useful to him would contain brief and clear accounts of diseases, and of the prevalent opinions concerning them, including notices of the appearances found after death, and of the general treatment. Such a work would be well suited to the first six months of his hospital attendance; and its outline should be filled up by the clinical lectures. As the student became more advanced, he should be referred to the best monographs on the subjects of cases of interest presented to his observation at the time. Without reading, the observation of a student is not very productive; but, combined with it, it ought to be a source of daily increasing knowledge. Dr. Latham is, we feel fully assured, very far above the affectation of that kind of originality the claim to which can only be supported by the exclusion of medical authors from any share of the attention of his hearers; but he appears to us not to be quite aware to what an extent medical reading is disregarded in the London school, and by those who come forth from it. The mind of a London student seldom seems trained to the attention required for reading an original work with a perfect accompanying of the author from his observations to his deductions. That which is almost alone thought worthy of perusal in any work, is the chapter on treatment; and to the fostering of this indolence of mind the clinical lectures even of celebrated men have, we apprehend, contributed. The apparent cleverness and clearness of observation, and the confident details of treatment which give life to such discourses, have alienated the student from the less attractive but more safe and substantial lessons to be gained from reading and reflecting on more finished productions. Anything falling short of certainty is regarded by the London student as indicative of some deficiency in his teacher. All that he demands is, to be taught what to do. He has no conception of being grateful for being taught how to think. This latter error, however, Dr. Latham has not encouraged; a great part of his lectures being devoted to teaching the student to observe and to draw conclusions for himself.

Dr. Latham's manner is often striking, as in the following passages, with which his sixth lecture commences.

"In going round the hospital, my mind often reverts to the time when I was a mere beginner like yourselves; and I remember how strange and puzzling to me was everything that I saw; how I thought I never should be able to distinguish diseases, one from another, as long as I lived; and, as to treating them, I could not look forward with the hope that my conscience would ever allow me to attempt any such thing. Above all, I was perplexed with the number and variety, and (as I humbly thought) contradictory nature of symptoms. It seemed to me, that if I could ever succeed in learning them all, it would be of no profit, for the same symptoms appeared sometimes to import one thing, and sometimes another. There was a patient, perhaps, suffering convulsions, and the physician evidently thought the case most grave and perilous, for he employed several remedies of the most gigantic power, and succeeded in saving him. But there was another patient suffering convulsions no less severe, and to my apprehension just of the same kind, yet so far was the physician from thinking seriously of this case, or treating it severely, that he just looked at the patient and smiled, ordered some cold water to be thrown in her face whenever the convulsions returned, and said that would cure her; and, sure enough, he was right. Moreover, many died who seemed to me to have little or nothing the matter with them, and many recovered whom I did not hesitate to condemn to death at first sight." (P. 140.)

Yet, although we admire the vivacity with which all this is expressed, we cannot but say, that, reflecting upon any experience of our own, the thoughts traced in the above passage appear to us to be such as would be far more likely to arise in the mind of a well-informed physician, than such as would offer themselves to the undisciplined student; and perhaps this peculiarity, which often gives to Dr. Latham's style in these lectures rather the appearance of being studied to produce an effect, arises from the difficulty with which any thing within his own experience can really suggest to him the state of mind of the majority of the students sent up from the country to the hospital with little previous education. There appears, too, a similar kind of inapplicability in avowing the extremest state of doubt as to the propriety of recommending any book on the practice of medicine, and yet advising the raw youth to animate his comprehension of the relative value of symptoms by the "careful perusal" of Abercrombie on the Intellectual Powers. Nor can we think the refinements which occur where Dr. Latham introduces the subject of symptoms are at all calculated to convey clear ideas to a student. They partake of the uselessness, as far as the mere learner is concerned, of all disquisitions on semeiology, although very large books have been compiled to illustrate that subject. They are like the clue to him who enters the labyrinth, of no use until its mazes have been explored, and he wishes to reconsider the steps that he has taken.

The modifications or changes in the economy induced by the slow and long-continued operation of stimulants present many curious circumstances to the attention of the pathologist; and the following description of their effect on the sensations derives, in our mind, additional interest from the analogy of such effect with the torpor and indifference to moral emotions and intellectual pleasures so often resulting from the same unhappy cause. The explanation of such changes is probably to be sought in the nervous system.

"I have often remarked, in the victims of extreme intemperance, that they have little or no consciousness of the pains and disordered sensations proper to the diseases which they suffer. This strange want of correspondence between the symptoms of disease derived from other sources, and those derived from the sensations, is a subject of very curious speculation medically, and of very melancholy interest morally. For the chief cause of the anomaly I believe to be really that to which I have alluded.

There are whole classes of society in London who are never really sober for years together. The sensations proper to health and to disease are alike unknown to them. In health, the stimulus of spirits, renewed day by day, and hour by hour, gives them feelings and excitement which are unnatural; and, however they may be mistaken for those of health, do in truth not at all belong to it. They are better, perhaps, and more pleasurable than any that health has to give, and they have superseded them. In disease (disease which it has itself produced), the stimulus of spirits gives them feelings and excitement which are still unnatural, and disguise or supersede the sensations which they then ought to have.

"People are frequently brought into the hospital just ready to perish of complicated visceral disease, yet declaring that they never suffered ache or pain in their lives until a few weeks ago. Their liver, spleen, kidney, and heart, and blood-vessels, are all disorganized. They are breathing, perhaps, with one lung; and the cellular structure and some cavities of the body are distended with fluid. Here is a disease which must have been the growth of years; yet true it is, as they say, that they have felt neither ache nor pain until within a few weeks. Spirits—spirits more and more recklessly taken—have sustained and excited, and cheated them, with false strength and false feelings, till fluid has gushed out everywhere, and vital organs have been suddenly oppressed, and down they have sunk at once, and irretrievably." (P. 151.)

These observations are not only interesting in themselves, and in relation to pathology, but deserve to be remembered by those who have to treat diseases as they occur in such habits. The application of principles of practice frequently requires caution, when what has been learned in one climate or country is acted upon in another; and, considered physiologically and pathologically, the inhabitants of a large city and of a small village can hardly be said to be of the same region.

About half of Dr. Latham's little work is devoted to the subject of auscultation, the neglect of which must be too well known to so experienced a London teacher. This neglect is so general that we cannot help ascribing it to a cause we have already alluded to,—a want of opportunities of learning how to apply the stethoscope, and a complete misconception as to what the ear is to expect when it is employed. To one who has listened half-a-dozen times to the natural sounds of the heart, and those which attend inspiration and expiration, some of the sounds which depart from those that are normal are so palpable, so easily recognised, and with so much certainty associated with particular pathological conditions, that, after once witnessing a good example of the latter kind, he cannot evermore doubt the value of the stethoscope. The knowledge of the morbid signs, thus commenced, is easily increased, until all the most useful indications are readily available to the practitioner. It may be that zealous auscultators, gifted with remarkable delicacy of ear, have increased the number and the nicety of these indications with the effect of discouraging those only commencing the practice of auscultation; and this consideration gives particular value to the instructions which Dr. Latham has laid before his pupils, with the intention of inciting them to employ their ears, in suitable cases, as continually and as effectually as their hands and their eyes. We must say, indeed, that he has delivered himself on this subject with so much judgment, clearness, and force of persuasion, that, for this reason, if for no other, his lectures would be usefully placed in the hands of every student. He sets out by explaining to them, with reference to the principal diseases of the chest,—pneumonia, pleurisy, and phthisis,—that there is no such thing as a pneumonic, a pleuritic, or a phthisical sound; but that the sounds heard in such diseases are the results of certain morbid processes going on, or certain changes wrought in the structure of the parts affected; that such sounds denote that a part of the lung is loaded with fluid, or condensed with solid matter, or hollowed into cavities; and that by this means "we anatomise by auscultation," and anticipate the disclosures of morbid anatomy. His recommendation first to study auscultation in healthy subjects is in strict accordance with the opinion already expressed by us; and he takes care, by some judicious remarks on the varieties of healthy respiration, to prevent discouragement to the beginner, which might arise from the various intensities of the respiration in various constitutions. In short, the eight lectures which Dr. Latham has devoted to the subject of auscultation, and which we shall not attempt to condense, deserve the most attentive perusal of the student, who should at the same time never visit the hospital without a stethoscope. It is only by the daily use of this instrument that it can be made fully available to the purposes of the practitioner. Dr. Latham has very candidly stated some of the difficulties and mistakes that are incidental to its application, but has also shown that they are such as may be overcome by patient repetition, and by careful reflection. The student, whom his admirable lessons encourage to make himself master of this instrument, will not only find that he has become an adept in the diagnosis of diseases of the chest, but that he has acquired a large fund of pathological knowledge, and a habit of exact examination which will daily add to its amount. A lecturer does his highest duty to his pupils when he thus prepares them. The pretender labours to instil his own peculiar doctrines; to create a party in favour of certain theories; to direct his followers to modes of practice that are to reflect upon the teacher the fame of originality. The man of science knows that the period of study is so short as not to permit the mind to become fully stored, or the opinions to be fully formed; he looks forward to the improved views which successive years surely bring, and his principal aim is so to inform and discipline the minds of his pupils as to make them safe and useful practitioners, and prepared to acquire whatever knowledge they have the means of obtaining throughout all the years of their practice. The tendency of Dr. Latham's lectures is precisely of this kind; and it is unnecessary for us to say more, either to recommend them to students, or to express our approbation of their merits.

